# Exosomes containing miR-148a-3p derived from mesenchymal stem cells suppress epithelial-mesenchymal transition in lens epithelial cells

**DOI:** 10.1093/stcltm/szae091

**Published:** 2025-02-26

**Authors:** Jingyu Ma, Qihang Sun, Yijia Chen, Jinyan Li, Shuyi Chen, Lixia Luo

**Affiliations:** State Key Laboratory of Ophthalmology, Zhongshan Ophthalmic Center, Sun Yat-Sen University, Guangdong Provincial Key Laboratory of Ophthalmology and Visual Science, Guangzhou, Guangdong 510060, People’s Republic of China; Department of Ophthalmology and Visual Sciences, The Chinese University of Hong Kong, Hong Kong 999077, People’s Republic of China; State Key Laboratory of Ophthalmology, Zhongshan Ophthalmic Center, Sun Yat-Sen University, Guangdong Provincial Key Laboratory of Ophthalmology and Visual Science, Guangzhou, Guangdong 510060, People’s Republic of China; Department of Ophthalmology, The Key Laboratory of Advanced Interdisciplinary Studies Center, The First Affiliated Hospital of Guangzhou Medical University, Guangzhou, Guangdong 510120, People’s Republic of China; State Key Laboratory of Ophthalmology, Zhongshan Ophthalmic Center, Sun Yat-Sen University, Guangdong Provincial Key Laboratory of Ophthalmology and Visual Science, Guangzhou, Guangdong 510060, People’s Republic of China; State Key Laboratory of Ophthalmology, Zhongshan Ophthalmic Center, Sun Yat-Sen University, Guangdong Provincial Key Laboratory of Ophthalmology and Visual Science, Guangzhou, Guangdong 510060, People’s Republic of China

**Keywords:** hucMSC-derived exosomes, LECs, EMT, miR-148a-3p, PRNP

## Abstract

Epithelial-mesenchymal transition (EMT) of lens epithelial cells (LECs) is responsible for the development of fibrotic cataracts, which contribute to severe visual impairment. Recent evidence has shown that mesenchymal stem cell-derived exosomes (MSC-Exo) can attenuate EMT in several tissues. However, the effect of MSC-Exo on EMT in LECs (LECs-EMT) has not been determined. In this study, we isolated exosomes from human umbilical cord MSCs (hucMSC-Exo) and evaluated their effect on LECs-EMT both in vitro and in vivo. HucMSC-Exo application significantly suppressed the expression of mesenchymal cell-associated genes while increasing the expression of epithelial cell-associated genes. Cell proliferation and migration of LECs undergoing EMT were inhibited after hucMSC-Exo treatment. The volume of EMT plaques in mice with injury-induced anterior subcapsular cataract (ASC) was significantly reduced in the hucMSC-Exo-treated group. Furthermore, miR-148a-3p was abundant in hucMSC-Exo. After transfection with miR-148a-3p inhibitor, the anti-fibrotic effect of hucMSC-Exo was attenuated in LECs-EMT. A dual-luciferase reporter assay identified PRNP as a direct target gene of miR-148a-3p. Furthermore, we verified that hucMSC-Exo inhibited LECs-EMT through the miR-148a-3p/PRNP axis and the potential downstream ERK signaling pathway. Taken together, our work reveals the inhibitory effect of hucMSC-Exo on LECs-EMT and the underlying mechanism involved, which may provide potential therapeutic options for fibrotic cataracts.

## Graphical Abstract

**Graphical Abstract:**
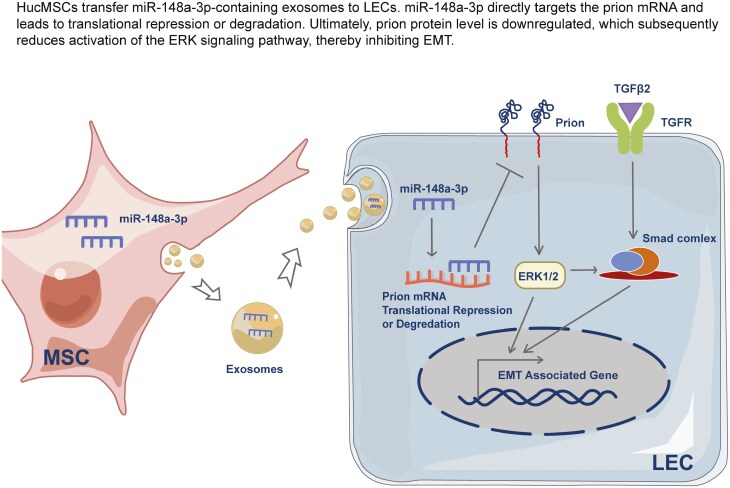

**Significance statement**

Posterior capsule opacification (PCO) is the most common long-term complication of cataract surgery and significantly affects the postoperative vision of many patients. The current lack of effective treatments highlights the need for improved therapeutic approaches. The major pathological change in PCO is the epithelial-mesenchymal transition (EMT) of lens epithelial cells (LECs). Our study indicates that hucMSC-Exo effectively suppresses LECs-EMT via the miR-148a-3p/PRNP axis and the downstream ERK signaling pathway, suggesting a promising therapeutic strategy for the treatment of PCO.

## Introduction

Fibrotic cataracts consist of posterior capsular opacification (PCO) and anterior subcapsular cataract (ASC). PCO, resulting from the residual lens epithelial cells (LECs), is the most common long-term complication of cataract surgery.^1^ Cataract is the leading cause of blindness and surgery remains the only effective treatment.^2^ A significant proportion of patients, in particular 20%-40% of adults^3^ and approximately 100% of children and infants,^4^ will develop PCO after surgery. Opacifying plaques can cause severe visual impairment, especially in patients with advanced intraocular lenses (IOLs). Even mild PCO can have a significant impact on visual quality.^5,6^ In addition, the most commonly used treatment for PCO, Neodymium:YAG laser (Nd:YAG) capsulotomy, has a high risk of complications.^7^ Therefore, research into new therapies and early treatment of PCO is imperative. In addition, ASC shares similar cellular and molecular mechanisms with PCO, which is the accumulation of fibrotic opacities beneath the anterior capsule caused by trauma, inflammation, or radiation,^8^ also poses a serious threat to vision.

The major pathological change in fibrotic cataracts is epithelial-mesenchymal transition (EMT).^9^ This process refers to the transformation of LECs from polygonal epithelial cells to spindle-shaped mesenchymal cells and is accompanied by enhanced migration and proliferation.^1^ Surgical injury and intraocular cytokine changes can induce the transdifferentiation of LECs into fibroblasts that express alpha-smooth muscle actin (α-SMA), vimentin, and secrete excessive fibronectin (FN) and collagen type I (Col I). Notably, TGFβ is the central cytokine-inducing EMT^10^ and TGFβ2 is the predominant subtype in the aqueous humor during lens EMT.^11^ In response to TGFβ2 or other stimuli, LECs migrate to the posterior capsule or anterior capsule and undergo EMT. These changes cause extracellular matrix deposition, capsular contraction, and subcapsular fibrotic plaques, ultimately leading to visual impairment.^11^ In addition, EMT is also the primary pathological process underlying fibrosis in various organs, including pulmonary fibrosis,^12^ hepatic fibrosis,^13^ renal fibrosis,^14^ and dermal fibrosis.^15^ Previous studies indicate that the lens is an excellent model for studying fibrosis due to its unique biological properties.^16^ Therefore, inhibition of LECs-EMT and understanding its underlying mechanisms may not only provide new strategies for the treatment of PCO but also provide new insights into the pathogenesis of fibrotic diseases.

Mesenchymal stem cell (MSC) therapy has recently emerged as a promising approach for the prevention and treatment of fibrotic diseases due to its potent tissue repair capabilities. MSCs have anti-fibrotic effects on liver^17^ and lung fibrosis.^18^ The therapeutic effects of MSCs are largely due to their paracrine factors, particularly through exosomes (Exo).^19^ Exosomes, nanometer-sized vesicles that mediate cellular communication, contain microRNAs (miRNAs), mRNA, and proteins from the source cells.^20^ With no risk of tumor formation and reduced immunogenicity, they offer great potential as novel alternatives to cell therapies with promising clinical applications.^21^ Several studies have shown that MSC-Exo have significant anti-fibrotic effects on liver fibrosis,^22^ the damaged endometrium,^23^ lipopolysaccharide (LPS)-induced acute lung injury,^24^ and renal tubular epithelial cells.^25^ Several clinical studies on MSC-Exo have been initiated.^26^ However, it remains unclear whether MSC-Exo can inhibit the EMT of LECs and be used for the treatment of PCO.

MiRNAs, a group of small non-coding RNAs, regulate gene expression post-transcriptionally by either degrading target mRNA or blocking translation.^27^ Several miRNAs, including miR-26,^28^ miR-22-3p,^29^ miR-497-5p,^30^ let-7a-5p,^31^ and miR-181a,^32^ have been found to inhibit EMT in LECs. However, the involvement of miR-148a-3p in lens fibrosis remains unknown. In our study, we found that miR-148a-3p was significantly downregulated in LECs after TGFβ2 treatment, suggesting its potential role as a suppressor of LECs-EMT. Considering that miRNAs are the major bioactive molecules in exosomes,^20^ we analyzed the miRNA expression profile of hucMSC-Exo and detected high levels of miR-148a-3p, suggesting its significant role in hucMSC-Exo function. Exosomes deliver miRNAs to the target cells to regulate various biological cellular processes. Studies have shown that miRNAs in MSC-Exo can suppress EMT. Exosomal miR-27b from human umbilical cord MSCs (hucMSC) was shown to attenuate subretinal fibrosis^33^; MSC-Exo delivered miR-23a-3p and miR-182-5p to halt the progression of LPS-induced lung fibrosis^24^; and miR-34c in MSC-Exo decreased invasion, migration, proliferation and EMT in nasopharyngeal carcinoma cells.^34^

In this study, we demonstrated that hucMSC-Exo inhibited TGFβ2-induced LECs-EMT both in vitro and in vivo. HucMSC-Exo mediated the inhibitory effect of LECs-EMT through the miR-148a-3p/PRNP axis and the potential downstream extracellular signal-regulated kinase (ERK) signaling pathway. These findings highlighted the suppressive effect of hucMSC-Exo on LECs-EMT and suggested a potential therapeutic strategy for the treatment of fibrotic cataracts.

## Materials and methods

### Cell culture

The human LEC line FHL124 was kindly provided by Professor David Wan-Cheng Li at the Zhongshan Ophthalmic Center.^35^ FHL124 cells were cultured in Dulbecco’s modified Eagle’s medium (DMEM, C11995500BT; Gibco) supplemented with 10% fetal bovine serum (FBS, Biowest) at 37°C, 5% CO_2_ and passaged by TrypLE (12605028; Gibco). To induce EMT, cells were seeded in plates (24-well or 12-well) and exposed to 10 ng/mL TGFβ2 (100-35B; PeproTech) for 48 hours.^36^

HucMSCs were purchased from Bioharbor (LH-C069). HucMSCs were cultured in DMEM (C11995500BT; Gibco) supplemented with 10% FBS (Biowest) at 37°C, 5% CO_2,_ and passaged with trypsin (25200072; Gibco). To verify the identity of the hucMSCs, flow cytometry was used to analyze the surface markers CD34, CD44, HLA-DR, CD45, CD90, and CD105 (BD Biosciences). To maintain hucMSCs, the cells were seeded in a petri dish, and the culture medium was changed every 48 hours. HucMSCs from passages 3 to 7 were used for the experiments. To prepare the conditioned supernatant for isolating exosomes, the serum-containing culture medium was removed when the cells reached 70% confluence. The cells were then rinsed with HBSS (14175095; Gibco), and the medium was subsequently switched to the serum-free hucMSC sEV secretion-promoting medium UltraCULTURETM (BP12-725F; Lonza). After 48 hours, the supernatant was collected.

### Isolation and identification of exosomes

Exosomes were extracted from the conditioned supernatant of hucMSCs using different centrifugation methods. After centrifuging the supernatant at 300×*g* for 10 minutes, the resulting supernatant was centrifuged again at 2000×*g* for 30 minutes to remove cell debris. After collection, the supernatant underwent centrifugation at 10 000×*g* for 60 minutes. The supernatant was subsequently collected and filtered through a 0.22 μm filter. The samples were then centrifuged at 100 000×*g* for 90 minutes (Optima XE 100, Beckman), the supernatant was discarded, and the pellet was resuspended in PBS. After another round of centrifugation at 100 000×*g* for 90 minutes, the resulting pellet was resuspended in 200 μL of PBS and then frozen at −80 °C for future use. The morphology of the hucMSC-Exo was determined by transmission electron microscopy (TEM). In brief, 50 μg of hucMSC-Exo was diluted (1:40) with PBS, then the exosomes suspension (20 μL) was poured onto a 300-mesh cell strainer for 10 min. After filtering out the extra liquid, the sample was counterstained with 1% phosphotungstic acid for 30 s and observed via TEM. Exosomes were analyzed for particle number and size distribution using Nanoparticle tracking analysis (NTA, NanoSight NS300). Western blot was used to analyze the exosome surface markers CD63 (1:1000), CD9 (1:1000), and CD81 (1:1000) (EXOAB-KIT-1; System Biosciences). The protein concentrations of the hucMSC-Exo were determined using a BCA Protein Assay Kit (A55864; Thermo Scientific).

### Exosome uptake assessment

To monitor the internalization of hucMSC-Exo into LECs, we first performed exosome labeling. After reconstituting the hucMSC-Exo (100 μg) in 1 mL of PBS, 10 µL of Dil Cell-Labeling Solution (V22885; Thermo Fisher) was added to the suspension. After gentle mixing, the suspension was incubated at 37 °C for 10 minutes. Afterward, the mixed suspension was then centrifuged at 100,000×*g* for 60 minutes. After the supernatant was discarded, the pellet was then resuspended in PBS to obtain Dil-labeled hucMSC-Exo. The labeled exosomes were then added to LECs in serum-depleted medium for 24 hours. For live-cell imaging, cells were washed 3 times with PBS, replaced with fresh DMEM, and then live Z-stack fluorescence images were collected using a Zeiss LSM980 confocal microscope (Carl Zeiss). LECs cocultured with Dil-labeled exosomes were also fixed with 4% paraformaldehyde for fluorescence imaging. Briefly, after washing the cells 3 times with PBS, the nuclei were counterstained with DAPI and then observed using the LSM980.

### Wound-healing assay

Once the LECs had grown to 90% confluence, the culture medium was replaced with serum-free medium to eliminate the influence of serum. After 12 hours of cell starvation, striae were made using a 200 μL micropipette tip. After removing the detached cells with PBS, the attached cells were cultured in serum-free DMEM with hucMSC-Exo (100 μg/mL) and TGFβ2 (10 ng/mL). After a 48-hour incubation, migration was monitored, and images were taken with an inverted phase-contrast microscope. ImageJ software (1.53a) was used to evaluate the wound area in each image. Experiments were performed with 5 biological replicates.

### EdU staining assay

The proliferation of LECs was assessed using the BeyoClick EdU Cell Proliferation Kit (C0071S; Beyotime) according to the manufacturer’s guidelines. In short, FHL124 cells were exposed to hucMSC-Exo (100 μg/mL) and TGFβ2 (10 ng/mL) for 48 hours. Following this, the culture medium was replaced with fresh basal medium containing 10 μM EdU for 4 hours. The cells were then fixed with 4% paraformaldehyde for 10 minutes, rinsed with PBS, and sequentially incubated with Click reaction buffer for 30 minutes. The cells were then stained with DAPI and then photographed using an LSM980 confocal microscope (Carl Zeiss).

### Mouse model of injury-induced ASC

All animal experiments were approved by the Animal Use and Care Committee of the Zhongshan Ophthalmic Center at Sun Yat-Sen University (Animal protocol No. Z2021016). Mice were housed in a standard environment with regular light/dark cycles and free access to water and a chow diet. Three- to four-weeks -old female and male C57BL/6J mice were used in our study. Injury-induced ASC in mouse eyes was generated as previously reported.^37^ Mice were anesthetized by intraperitoneal injection of pentobarbital sodium (70 mg/kg) and topical application of dicaine eye drops. The pupil was dilated with tropicamide eye drops. A small incision was then made in the central anterior capsule of the eye through the cornea using the inclined plane of a 26-gauge hypodermic needle. The wound depth was approximately 300 μm which is about a quarter of the length of the needle blade. The direction of the needle insertion was perpendicular to the center of the cornea. Immediately after injury, 2 μL of hucMSC-Exo (2 μg/μL) were injected into the anterior chamber of the eye using a microsyringe (30-gauge, Hamilton), while the control eye received the same volume of saline. In each experiment, the depth and angle of needle insertion were kept consistent to ensure uniform incisions. The same operator performed this part of the experiment each time. After the surgery, tobramycin eye ointment was applied to prevent infection. Seven days after surgery, the anterior lens capsules were harvested for whole-mount staining. To assess exosome uptake in vivo, the same amount of Dil-labeled exosomes was injected into the anterior chamber. The anterior lens capsule was harvested for whole mount staining, and images were collected using an LSM980 confocal microscope (Carl Zeiss).

### Whole-mount staining of the anterior lens capsule and analysis of plaques

Injured mice were euthanized, and the lenses were isolated. Next, the lenses were fixed in 100% methanol for 1 hour at room temperature (RT). The anterior capsules of the lenses were then separated and treated with a blocking solution containing 1% Triton X-100 in 5% normal donkey serum for 1 hour at RT. Primary antibodies against FN (ab137720; Abcam) and α-SMA (ab5694; Abcam) were added to the capsules, which were then incubated overnight at 4 °C. After PBST rinses, the capsules were incubated with the appropriate secondary antibodies for 1 hour at RT. After DAPI counterstaining, the whole anterior capsules were mounted on a microscope slide using anti-fade medium. Z-stack images of the whole anterior subcapsular plaque were collected with an LSM980 confocal microscope.

The quantitative method for measuring plaque volume was based on published methods.^38^ In this model, the subcapsular plaque resembles the shape of a pyramid’s frustum. Therefore, the subcapsular plaque volume in each image pair was calculated using the formula for pyramid volume: $V1\;\;\;=\;\;\;1/3\;\;\;\times \;\;\;H\;\;\;\times \;\;\;[S_{up}+S_{down}+\sqrt{S_{up}\times S_{down}}$]. The total volume of the subcapsular plaque in each sample was the sum of the volumes from each image pair (*V*_total_ = *V*_1_ + *V*_2_ +... + *V*_*n*_). At least 8 capsules were analyzed in each group.

### Transfection of the miR-148a-3p mimic and inhibitor

To analyze the role of miR-148a-3p, miRNA mimics or inhibitors were used to enhance or suppress the expression of miR-148a-3p, respectively. For overexpression of miR-148a-3p, a mixture of 100 nM mimic negative control (mi-NC) or miR-148a-3p mimic (RiboBio) in combination with riboFECT CP buffer (C10511-05, RiboBio) was added to the LECs at 60%-70% confluence in DMEM. In addition, for miR-148a-3p knockdown, the cells were transfected with 150 nM inhibitor negative control (inh-NC) or miR-148a-3p inhibitor (RiboBio). After 4–6 h incubation, the medium was replaced and 10 ng/mL of TGFβ2 was added for a further 48 hours. The sequences of miR-148a-3p inhibitor, inhibitor NC, miR-148-3p mimic, and mimic NC are listed in additional file 1 (Supplementary Table S1).

### Dual luciferase reporter assay

To confirm whether PRNP was the direct target of miR-148a-3p, a luciferase reporter assay was performed. The PRNP sequence with either the wild-type (wt) or mutant (mut) miR-148a-3p binding sites was cloned downstream of the firefly luciferase gene in the pGL3 luciferase reporter vector. The PRNP wt vector (PRNP-wt) or mutant vector (PRNP-mut) was co-transfected with either the miR-148a-3p mimic or mimic-NC, respectively. After 48 hours of incubation, the cells were harvested, and luciferase activity was measured using the Dual-Glo luciferase assay system.

### RNA isolation and quantification

Total RNA was extracted from cells using TRIzol reagent (15596-018; Thermo Fisher Scientific). Reverse transcription was performed using a PrimeScript RT Master Mix kit (TaKaRa), and quantitative PCR was performed using a SYBR Premix Ex Taq kit (TaKaRa) on a LightCycler 480 (Roche). GAPDH was used as an internal control. A miRcute miRNA isolation kit (DP501; Tiangen) was used for miRNA extraction. The cDNAs of the miRNAs were synthesized using a miRcute Plus miRNA First-Strand cDNA Kit (KR211; Tiangen), and the levels of the miRNAs were quantified via real-time PCR using a miRcute Plus miRNA qPCR Kit (FP411; Tiangen). U6 was used as an internal control. Relative gene expression was determined by the 2^−ΔΔCt^ method. The primers used in this study are listed in additional file 1 (Supplementary Table S2).

### Western blot analysis

For Western blot analysis, cells were harvested and lysed with RIPA buffer. The protein concentration was determined using a BCA protein assay kit (A55864; Thermo Scientific). The proteins were mixed with 5× SDS sample buffer, and equal amounts of protein were then separated via sodium dodecyl-polyacrylamide gel electrophoresis (SDS-PAGE) and electroblotted onto polyvinylidene fluoride (PVDF) membranes. After blocking with 5% nonfat milk, the membranes were incubated overnight at 4 °C with different primary antibodies. After washing with TBS containing 0.1% Tween 20 (TBST), the membranes were incubated with HRP-conjugated secondary antibodies. The target proteins were detected using an enhanced chemiluminescence reagent. The antibodies used included: antibodies against FN (ab137720; Abcam); α-SMA (ab5694; Abcam); Col I (ab138492; Abcam), Col IV (ab6586; Abcam), Snail (3879; Cell Signaling Technology, Massachusetts), vimentin (PTM-5376; PTMBIO), PRNP (14025; Cell Signaling Technology), p-ERK (4370S; Cell Signaling Technology), t-ERK (4696S; Cell Signaling Technology), and HSP90 (13171-1-AP; Proteintech).

### Immunofluorescence staining

The cells were fixed with 4% paraformaldehyde for 10 minutes at RT and then permeabilized with 0.5% Triton X-100 for 10 minutes. The cells were then blocked with 5% normal donkey serum for 30 minutes. The cells were then incubated overnight at 4 °C with various primary antibodies, including FN (ab137720; Abcam) and α-SMA (ab5694; Abcam). The next day, Alexa Fluor 488-conjugated or Alexa Fluor 568-conjugated secondary antibodies were applied and incubated for 1 hour at RT. After PBST washes, the cells were counterstained with DAPI for nuclear staining. Cells were observed using an LSM980 confocal microscope.

### Statistical analysis

All experiments were performed in at least 3 replicates. Data analysis was performed using GraphPad Prism version 8 (GraphPad Software Inc.). Group comparisons were performed using either Student’s *t* test for 2 groups or 1-way ANOVA followed by Tukey’s post hoc test for multiple group comparisons. All quantitative data are presented as mean ± SEM. *P* values less than .05 were considered statistically significant.

## Results

### Identification of hucMSCs and hucMSC-derived exosomes

**Figure 1.**
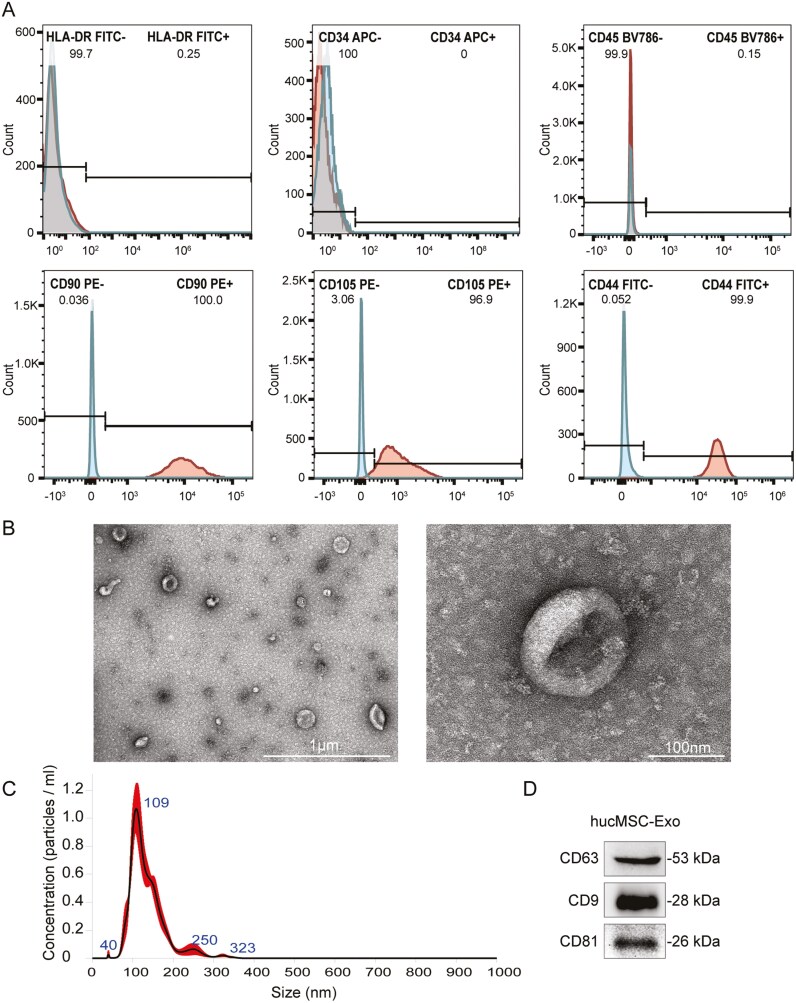
Identification and characterization of hucMSC-derived exosomes. A. Flow cytometric analysis of the surface markers of hucMSCs. The cells were positive for CD90, CD105, and CD44, and negative for HLA-DR, CD34, and CD45. B. TEM analysis of typical hucMSC-Exo. C. The relationship between the size and concentration of exosomes was measured by NTA (*n* = 3) D. The specific surface markers (CD63, CD9, and CD81) of the exosomes were evaluated by Western blot.

HucMSCs were characterized by flow cytometry. MSC markers (CD90, CD105, and CD44) were highly expressed, whereas non-mesenchymal stem cell markers (HLA-DR, CD34, and CD45) were expressed at extremely low levels on the cell surface (Figure 1A). We then isolated exosomes from the supernatant of the hucMSCs by ultracentrifugation. Transmission electron microscopy (TEM) confirmed that the exosomes were typically cup-shaped (Figure 1B). Nanoparticle tracking analysis (NTA) revealed that the sizes of most of the particles ranged from 30 to 150 nm, with a median size of 109 nm (Figure 1C). Western blot analysis of the vesicles from hucMSCs revealed the expression of the specific exosome markers, CD9, CD63, and CD81 (Figure 1D). Thus, TEM, NTA, and Western blot analyses confirmed the typical properties of hucMSC-Exo.

### HucMSC-Exo could be internalized into LECs and attenuate TGFβ2-induced EMT

**Figure 2.**
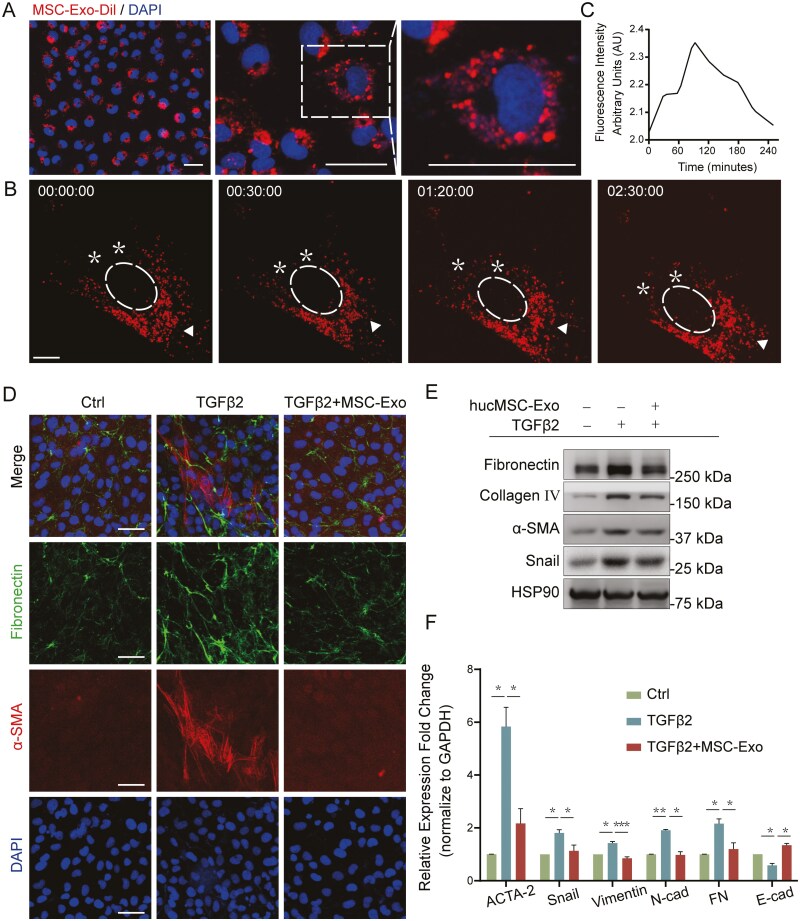
HucMSC-Exo were internalized into LECs and attenuated TGFβ2-induced EMT. A. Confocal images of FHL124 cells co-cultured with Dil-labeled hucMSC-Exo for 48 hours. Scale bar = 50 μm. B. Confocal images showing the time course of Dil-labeled hucMSC-Exo uptake by the LECs. Dashed lines indicate nuclei. Asterisks and triangles indicate soma regions where the fluorescence intensity of exosomes changed significantly with time. Scale bar = 10 μm. C. Time course of the fluorescence intensity over time in the asterisked region of Figure 2B. D. Immunofluorescence analysis of EMT markers in LECs after treatment with hucMSC-Exo and TGFβ2 for 48 hours. Scale bar = 50 μm. E. Western blot analysis of EMT-associated proteins in LECs after treatment with hucMSC-Exo and TGFβ2 for 48 hours. F. Real-time PCR analysis of EMT-associated gene expression in LECs after treatment with hucMSC-Exo and TGFβ2. The ACTA-2 gene encodes the α-SMA protein. *P < .05, **P < .01, and ***P < .001.

To investigate the effect of hucMSC-Exo on EMT, we first examined whether the exosomes could be internalized by LECs. The hucMSC-Exo were labeled with a membrane labeling dye (Dil), which specifically integrates into the membrane bilayer structure upon fusion. The labeled exosomes were co-cultured with LECs for 48 hours. Fluorescence microscopy analysis revealed the presence of red fluorescence in the cytoplasm of LECs (Figure 2A). We further analyzed the time course dynamics of cells engulfing exosomes. The results showed that the number of Dil-labeled exosomes in the soma of the LECs changed over time (Figure 2B and C), indicating that hucMSC-Exo could be taken up by LECs.

We treated LECs with TGFβ2 to establish an EMT model in vitro. Immunofluorescence and Western blot analysis verified that TGFβ2 treatment increased the expression of FN, α-SMA, collagen IV (Col IV), vimentin, and the transcription factor Snail (Figure 2D-F). Strikingly, hucMSC-Exo treatment reversed the TGFβ2-induced increase in the levels of α-SMA, FN, Col IV, vimentin, and Snail (Figure 2D-F). Using real-time PCR, we verified that the expression of EMT-associated genes was downregulated, while the expression of E-cadherin was upregulated in the hucMSC-Exo-treated group (Figure 2F). These results suggested that hucMSC-Exo suppressed TGFβ2-induced EMT in LECs.

### HucMSC-Exo inhibited extracellular matrix deposition, LEC proliferation, and migration

**Figure 3.**
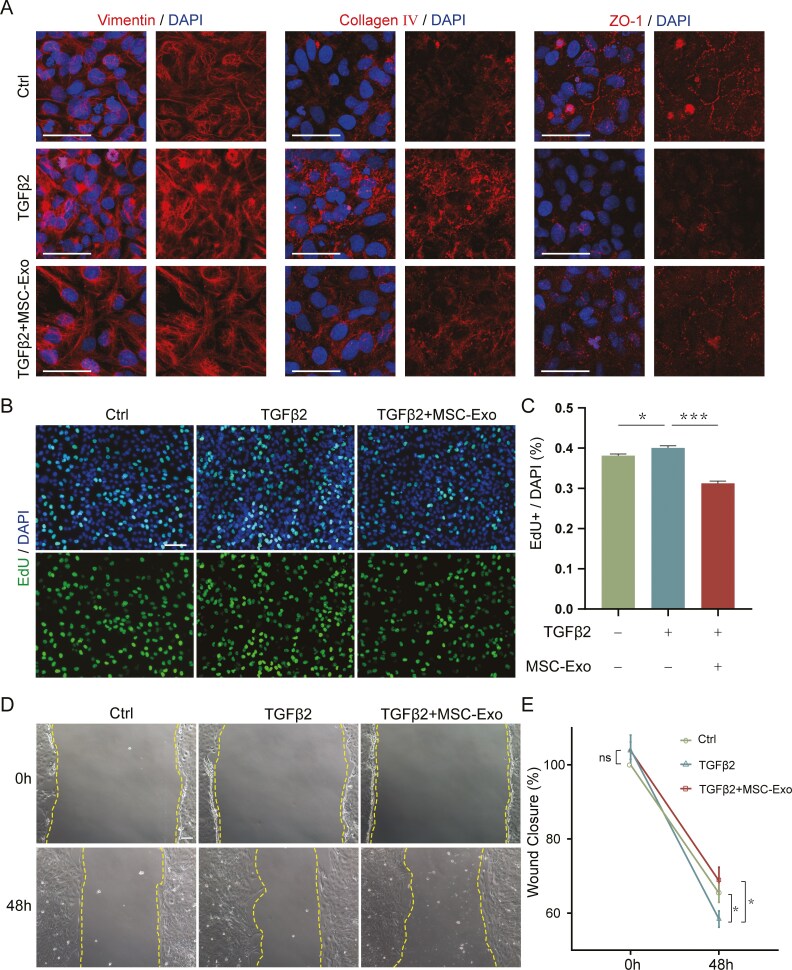
HucMSC-Exo inhibited extracellular matrix deposition, LECs proliferation, and migration. A. Immunofluorescence analysis of the extracellular matrix protein Col Ⅳ, the mesenchymal cell marker vimentin, and the epithelial cell marker ZO-1 in LECs after treatment with TGFβ2 and hucMSC-Exo. Scale bar = 50 μm. B. An EdU assay was used to analyze the proliferation of LECs after treatment with TGFβ2 and hucMSC-Exo. Scale bar = 100 μm. C. Quantification of the EdU^+^/DAPI ratio (%). *P < .05 and ***P < .001. D. Wound healing analysis of LEC migration after treatment with TGFβ2 and hucMSC-Exo for 48 hours. Scale bar = 100 μm. E. Quantification of the remaining wound area per field. *n* = 5; *P < 0.05; ns, not significant.

Since EMT is characterized by loss of epithelial characteristics, deposition of extracellular matrix, and increased cell proliferation and migration, we further investigated the effect of hucMSC-Exo on these processes. Immunofluorescence staining revealed that the TGFβ2-induced loss of epithelial characteristics was accompanied by an increase in the level of vimentin, a marker of mesenchymal cells, and a decrease in the level of ZO-1, a marker of epithelial cells. Notably, the application of hucMSC-Exo suppressed both the increase in vimentin expression and the decrease in ZO-1 expression (Figure 3A). Furthermore, TGFβ2 triggered the accumulation of extracellular matrix proteins, but co-culture with hucMSC-Exo significantly reversed the TGFβ2-mediated deposition of both FN and Col IV (Figure 2D and 3A). EdU staining showed that TGFβ2 treatment promoted the proliferation of LECs (Figure 3B). Interestingly, hucMSC-Exo significantly suppressed the TGFβ2-induced increase in proliferation of LECs (Figure 3B and C). Furthermore, a wound-healing assay showed that TGFβ2 enhanced the migration of LECs. However, the application of hucMSC-Exo dramatically suppressed the TGFβ2-induced increase in migration of LECs (Figure 3D and E). Collectively, these findings further demonstrated that hucMSC-Exo treatment significantly inhibited TGFβ2-induced LECs-EMT.

### HucMSC-Exo attenuated EMT in the injury-induced ASC mouse model

**Figure 4.**
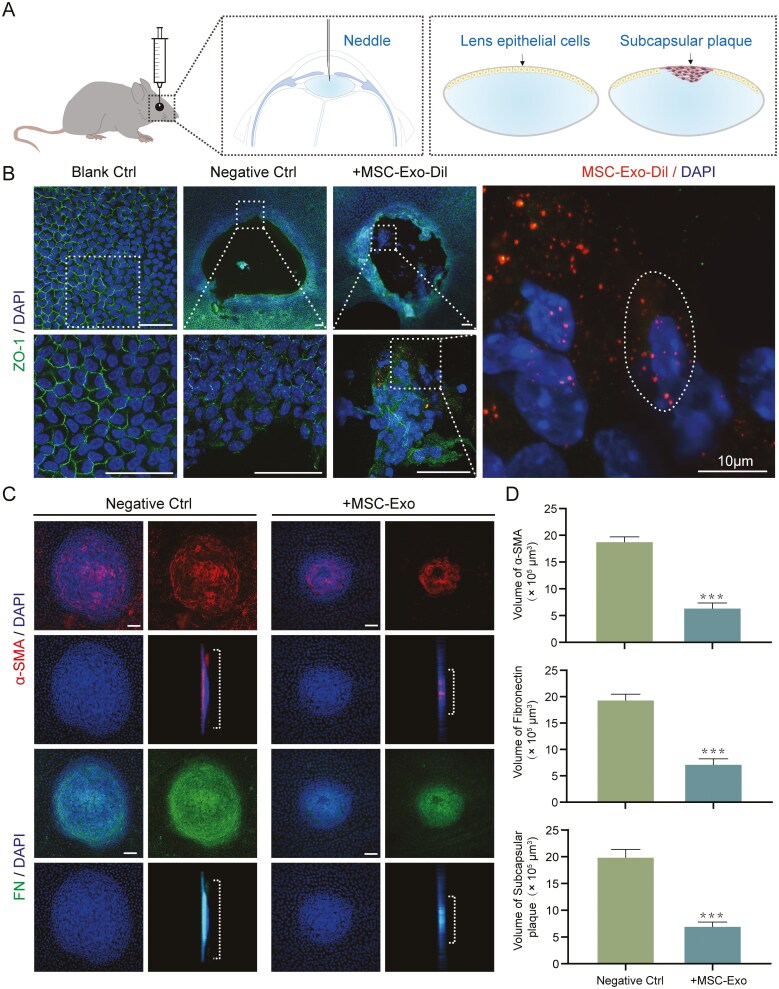
HucMSC-Exo ameliorated EMT in the injury-induced mouse model of ASC. A. Schematic diagram of the injury-induced ASC mouse model. B. The morphology of LECs 2 days after injury. Polygonal LECs were observed in the blank control group, and the shape of the injured area was observed in the negative control group. Scale bar = 50 μm. Exosomes were observed in the abnormal LECs of the group that received anterior chamber injection of Dil-labeled hucMSC exosomes. The dashed lines represent the outline of the cells. Scale bar = 10 μm. C. Images of whole mounts of the lens capsule showing areas of subcapsular plaque, along with α-SMA and FN staining at 7 days post injury. Scale bar = 50 μm. D. Volume quantitation of the subcapsular plaques. *n* = 8, ***P < 0.001. Partial illustrations were created using templates from www.motifolio.com.

We next examined whether hucMSC-Exo has similar effects in vivo. After puncture injury to the anterior lens capsule, the reparative processes involving cell proliferation and migration in epithelial cells resemble the process of EMT. The volume of subcapsular plaques can reflect the severity of EMT to some extent and is a well-established model for studying the EMT of LECs in vivo^28,35,39,40^ (Figure 4A). The morphologies of the normal anterior subcapsular LECs and LECs after 2 days of injury are shown in Figure 4B. To evaluate the ability of cells to take up hucMSC-Exo, we injected Dil-labeled exosomes into the anterior chamber of mice immediately after the injury. Two days after injection, red fluorescent signals were found in the cytoplasm of the LECs, especially those abnormal LECs that lacked the characteristics of epithelial cells, indicating that the LECs were able to take up hucMSC-Exo (Figure 4B). Seven days after surgery, a distinct white opaque plaque formed in the anterior capsule. Whole-mount staining of the anterior capsule of the lens revealed plaques with high levels of α-SMA and FN. Similarly, the volume of α-SMA, FN, and DAPI positive plaques was significantly reduced after hucMSC-Exo treatment compared to the negative control group (Figure 4C and D). Taken together, these results demonstrated the inhibitory effect of hucMSC-Exo on LECs-EMT in vivo.

### Analysis of the hucMSC-Exo miRNA profile and miR-148a-3p expression in hucMSC-Exo

**Figure 5.**
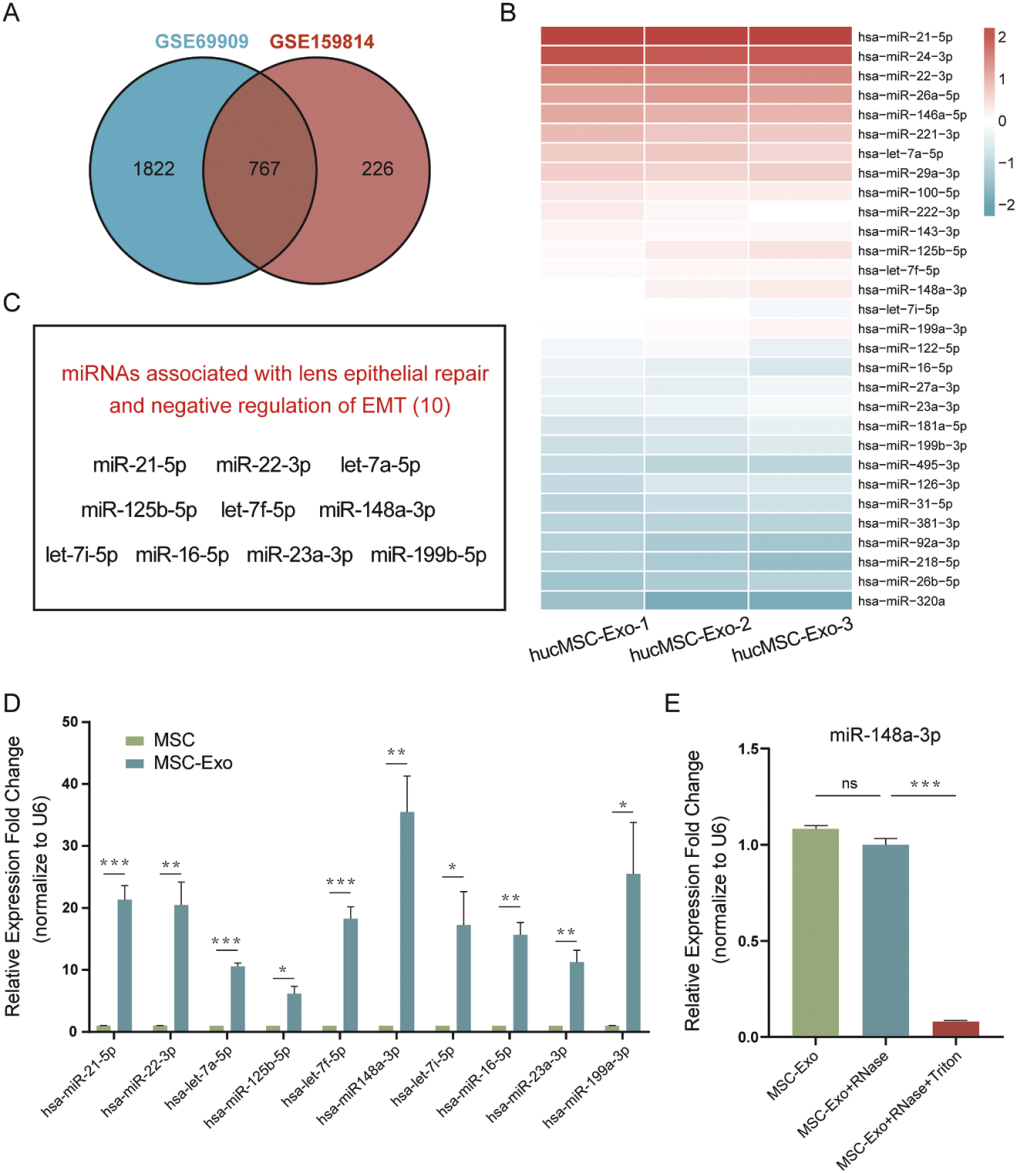
The miRNA expression profiles of hucMSC-Exo and the expression of miR-148a-3p in exosomes. A. Identification of conserved miRNAs in hucMSC-Exo using 2 different GSE datasets; the overlap of the miRNAs is shown in a Venn diagram. B. Heatmap of the 30 miRNAs with the highest expression levels. C. Ten candidate miRNAs associated with lens epithelial repair and negative regulation of EMT. D. Real-time PCR analysis of the 10 candidate miRNAs in hucMSC-Exo. *P < .05, **P < .01, and ***P < .001. E. The levels of miR-148a-3p in hucMSC-Exo were assessed using real-time qPCR after treatment with RNase and Triton X-100 for 30 minutes. ***P < .001; ns, not significant.

Numerous studies have shown the abundance of miRNAs in exosomes, suggesting their crucial role in mediating intercellular communication. To further investigate the specific molecular mechanisms through which exosomes inhibit EMT in LECs, we analyzed the miRNA expression profile of hucMSC-Exo using 2 GSE datasets (GSE69909 and GSE159814).^41,42^ By taking the intersection, we obtained a set of 767 miRNAs that were found to be conserved in hucMSC-Exo (Figure 5A). A heatmap of the 30 miRNAs with the highest expression levels is shown in Figure 5B. Among them, 10 miRNAs were found to be potentially involved in repairing LECs and negative regulation of EMT based on literature review (Figure 5C). By validating the levels of these miRNAs by real-time PCR, we found that miR-148a-3p had the highest level in hucMSC-Exo (Figure 5D). To further confirm the presence of miR-148a-3p in exosomes, we then determined the miR-148a-3p levels in hucMSC-Exo after treatment with RNase and 0.1% Triton X-100 for 30 minutes. The group treated with RNase alone did not show a significant decrease in the miR-148a-3p level unless co-treated with Triton, which can be attributed to Triton disrupting the exosome membrane, leading to miRNA degradation by RNase (Figure 5E). Taken together, these results indicated that miR-148a-3p was enriched in hucMSC-Exo and may be a key mediator of the inhibitory effect on LECs-EMT.

### miR-148a-3p inhibited TGFβ2-induced LECs-EMT

**Figure 6.**
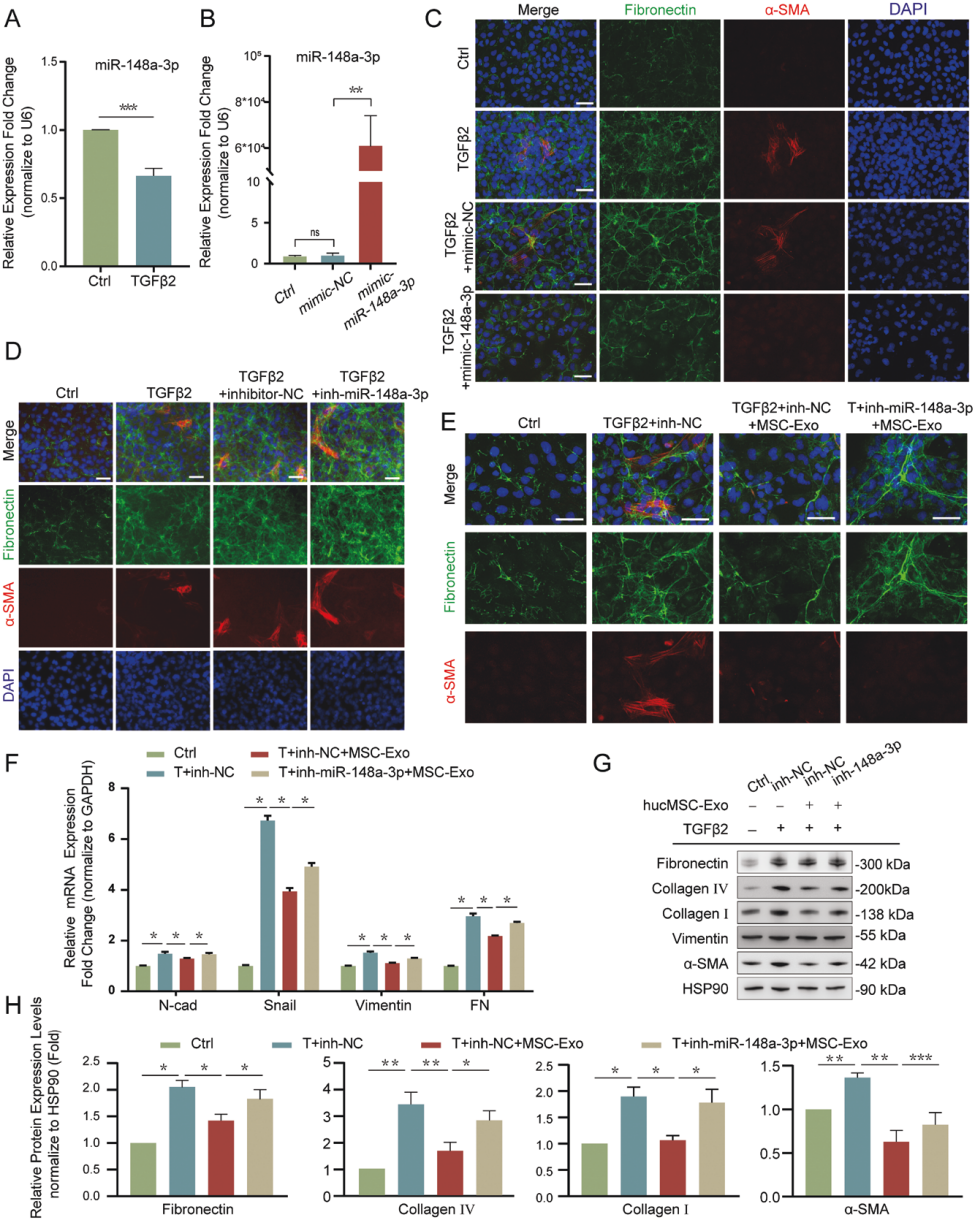
The inhibitory effect of hucMSC-Exo on EMT was attenuated after application of the miR-148a-3p inhibitor. A. The expression of miR-148a-3p was downregulated after TGFβ2 treatment. ***P < .001. B. Analysis of the transfection efficiency of the miR-148a-3p mimic by qPCR. **P < .01; ns, not significant. C. Immunofluorescence staining analysis of FN and α-SMA in LECs that were transfected with the miRNA mimic negative control (mimic-NC) or the miR-148a-3p mimic, and then treated with TGFβ2 for 48 hours. Scale bar = 50 μm. D. Immunofluorescence staining analysis of FN and α-SMA in LECs transfected with inhibitor negative control (inhibitor-NC) or miR-148a-3p inhibitor, followed by treatment with TGFβ2 for 48 hours. Scale bar = 50 μm. E. Immunofluorescence results showing FN and α-SMA expression in LECs after treatment with hucMSC-Exo and transfection with the miR-148a-3p inhibitor. Scale bar = 50 μm. F. Real-time PCR analysis of the expression levels of EMT markers in LECs after the application of hucMSC-Exo and transfection with the miR-148a-3p inhibitor. *P < .05. G. Effect of hucMSC-Exo and the miR-148a-3p inhibitor on EMT-related proteins assessed by Western blot. H. Quantification of EMT-related protein expression levels assessed by Western blot. *P < .05; **P < .01, and ***P < .001.

To determine the biological roles of miR-148a-3p in TGFβ2-induced EMT, we initially examined its levels in TGFβ2-treated LECs and found a notable decrease compared to the control group (Figure 6A), indicating that miR-148a-3p may be involved in the EMT process. Next, we performed gain- and loss-of-function experiments by transfecting LECs with a miR-148a-3p mimic and inhibitor oligonucleotides. Real-time PCR confirmed the transfection efficiency of the miR-148a-3p mimic (Figure 6B). Strikingly, the immunofluorescence results showed that overexpression of miR-148a-3p significantly downregulated the expression of EMT-related markers FN and α-SMA, whereas knockdown of miR-148a-3p upregulated these markers (Figure 6C and D). These data suggest that miR-148a-3p plays an inhibitory role in TGFβ2-induced LECs-EMT.

### The inhibitory effect of hucMSC-Exo on EMT was attenuated after knockdown of miR-148a-3p

To explore the effect of miR-148a-3p in hucMSC-Exo on TGFβ2-induced EMT, LECs treated with hucMSC-Exo were transfected with a miR-148a-3p inhibitor. Immunofluorescence revealed that transfection with the miR-148a-3p inhibitor led to the upregulation of FN and α-SMA compared to hucMSC-Exo treatment alone (Figure 6E). Real-time PCR also confirmed that EMT-associated genes were upregulated in hucMSC-Exo-treated cells when these cells were transfected with the miR-148a-3p inhibitor (Figure 6F). Furthermore, Western blot analysis showed that the levels of FN, Col IV, Col I, vimentin, and α-SMA were decreased after hucMSC-Exo treatment, while these effects were attenuated when the LECs were additionally transfected with the miR-148a-3p inhibitor (Fig. 6G and H). Therefore, these data confirmed that delivery of miR-148a-3p to LECs is one of the mechanisms by which hucMSC-Exo suppress TGFβ2-induced EMT.

### HucMSC-derived exosomal miR-148a-3p inhibited the EMT process by suppressing the PRNP and downstream ERK signaling pathway

**Figure 7.**
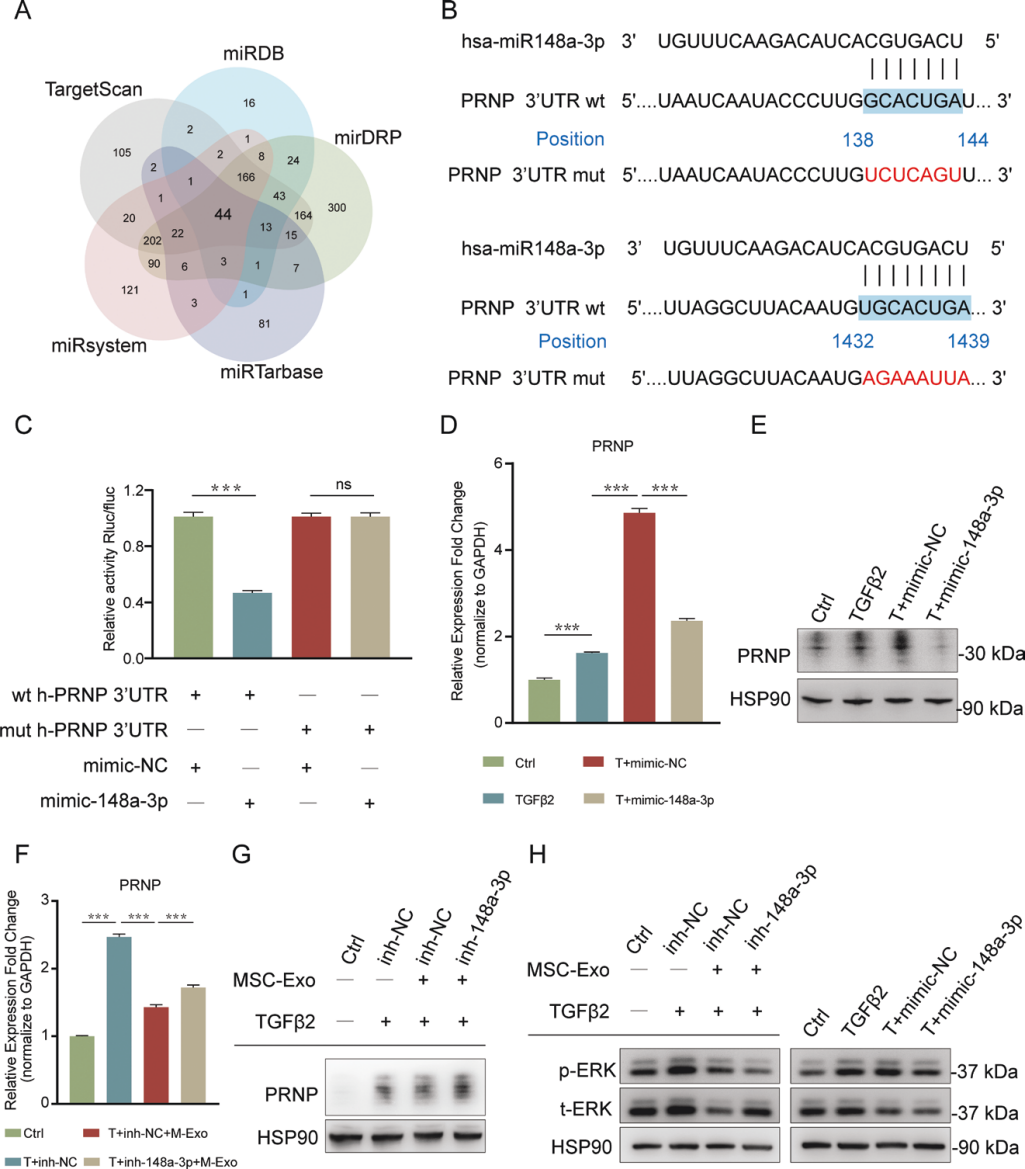
HucMSC-derived exosomal miR-148a-3p inhibited the EMT process by suppressing the PRNP and the downstream ERK pathway. A. Potential target genes of miR-148a-3p were predicted using 5 databases (TargetScan, miRanda, miRTarBase, miRSystem, and miRDB). B. The 3ʹUTR of the PRNP gene was complementary to miR-148a-3p C. A dual-luciferase reporter assay was performed to validate PRNP as a target gene of miR-148a-3p. ***P < .001; ns, not significant. D. Real-time PCR analysis of PRNP expression in LECs after transfection with the mimic negative control or the miR-148a-3p mimic. ***P < .001. E. Western blot analysis of PRNP expression in LECs after transfection with the mimic negative control or the miR-148a-3p mimic. F. Real-time PCR analysis of PRNP expression in LECs after the application of hucMSC-Exo and transfection with the miR-148a-3p inhibitor (inh-148a-3p). ***P < .001. G. The effects of hucMSC-Exo and the miR-148a-3p inhibitor on the expression of PRNP were assessed by Western blot analysis. H. Western blot analysis was performed to examine the effect of hucMSC-Exo, the miR-148a-3p inhibitor, and the miR-148a-3p mimic on the expression of p-ERK and t-ERK.

To search for the target genes of miR-148a-3p, we performed bioinformatics analysis with 5 target gene prediction databases (TargetScan, miRDB, mirDRP, miRsystem, and miRTarBase) online. The prediction data from the 5 databases were intersected, and 44 candidate target genes were identified (Figure 7A). Among them, PRNP exhibited the most significant change and was correlated with miR-148a-3p expression levels. Given that previous studies have revealed the involvement of PRNP in promoting the EMT process,^43,44^ we speculated that PRNP could be a potential target gene of miR-148a-3p. TargetScan predicted 2 miR-148a-3p binding sites in the 3ʹ untranslated region (UTR) of the PRNP gene (Figure 7B). In addition, a dual-luciferase reporter assay was used to confirm whether PRNP is a target of miR-148a-3p. The results showed that the luciferase activity of pSICheck-2-PRNP-wt was inhibited by the overexpression of miR-148a-3p, whereas the luciferase activity of pSICheck-2-PRNP-mut was unaffected (Figure 7C). These data indicated that miR-148a-3p can directly target PRNP. This finding was further confirmed by real-time PCR and Western blot. Overexpression of miR-148a-3p via transfection with the miR-148a-3p mimic significantly downregulated PRNP expression (Figure 7D and E) These results demonstrated that PRNP is a direct target gene of miR-148a-3p.

The PRNP gene encodes the prion protein (PrP^C^). PrP^C^ can interact with its ligand laminin to activate the ERK signaling pathway, thereby promoting EMT.^43^ PrP^C^ is expressed in the LECs as well.^45^ Additionally, ERK signaling was found to be required to mediate TGFβ2-induced LECs-EMT.^46^ Therefore, we examined the expression of PRNP and ERK in our study. The results showed that after TGFβ2 treatment, the ERK signaling pathway was significantly activated, consistent with the upregulation of PRNP expression (Figure 7F-H). These findings suggested that PrP^C^/ERK signaling pathway may be involved in LECs-EMT. To investigate the role of PRNP/ERK signaling in MSC-Exo-mediated inhibition of LECs-EMT, we evaluated the expression levels of p-ERK, t-ERK, and PrP^C^ after hucMSC-Exo treatment and miR-148a-3p knockdown. As expected, real-time PCR results showed that hucMSC-Exo treatment inhibited PRNP expression levels, while co-transfection with the miR-148a-3p inhibitor upregulated PRNP expression (Figure 7F). Consistent with this, WB results showed that after hucMSC-Exo treatment, PRNP, phospho-ERK, and total ERK expression were suppressed. However, co-transfection with the miR-148a-3p inhibitor significantly upregulated PRNP and total ERK expression (Figure 7G and H**),** suggesting that the inhibitory effect of hucMSC-Exo on the PrP^C^/ERK pathway was partially blocked. In addition, consistent with the trends observed for PRNP expression, ERK expression was significantly decreased after transfection with the miR-148a-3p mimic (Figure 7E and 7H). Taken together, these results showed that the inhibition of the EMT process by hucMSC-derived exosomal miR-148a-3p was achieved through the suppression of the PRNP/ERK signaling pathway.

## Discussion

In this study, we showed that hucMSC-Exo administration inhibited LECs-EMT. We found that miR-148a-3p in the exosomes plays an important role in the regulation of EMT. Our results showed that exosomal miR-148a-3p exerted its regulatory effects by inhibiting the PRNP expression and downstream ERK signaling pathways in the FHL124 cell line. Notably, xenogeneic human MSC-derived exosomes also effectively alleviated injury-induced ASC in mice.

PCO significantly affects the visual prognosis of patients who undergo cataract surgery.^47^ The Nd:YAG capsulotomy is associated with many complications including IOL optic impairment, transient intraocular pressure elevation, cystoid macular edema, and increased risk of retinal detachment.^47^ There is a need to explore optimized therapeutic strategies. In recent years, the modification and design of IOLs have shed light on PCO treatment.^48^ The design of IOLs focuses on sharper square edges to physically block the migration of LECs, thus preventing PCO. Surface modifications have also been explored to enhance this effect,^49^ but studies indicate that IOLs modifications are unlikely to significantly improve the barrier function.^50^ As a result, drug-loaded IOLs may offer a better option for treating PCO.^1^ Researchers have investigated loading cytotoxic drugs such as thapsigargin^51^ to eliminate residual LECs. However, the inevitable side effects of these drugs on surrounding tissues have hindered their further development.^52^

Exosomes have the advantage of cell targeting and serve as excellent molecular carriers.^20^ Therefore, combining exosomes with IOLs may be a viable strategy. Research has shown that the antiproliferative drug Dox can be encapsulated in LEC-Exo (Dox@Exos-IOLs), and Dox@Exos-IOLs exhibited better inhibitory effects on PCO in animal experiments.^53^ Our results revealed that hucMSC-Exo inhibited LECs-EMT, highlighting the potential of MSC-Exo-loaded IOLs as a stand-alone strategy for the prevention of PCO. Moreover, MSC-Exo also shows promise as a drug carrier on IOLs, improving drug targeting and therapeutic efficacy. However, when it comes to application, what is the best way to deliver hucMSC-Exo, how long hucMSC-Exo treatment affect LECs, whether the antifibrotic effect of MSC-Exo is dose-dependent, and whether drug-loaded MSC-Exo enhances its antifibrotic effects need to be further investigated.

MiRNAs are abundant exosomes cargoes that mediate their effects.^20^ Our study indicated that miR-148a-3p was one of the most abundant miRNAs in hucMSC-Exo based on microarray analysis, and qPCR further confirmed that it had the highest expression among the candidate miRNAs. MiR-148a-3p, part of the miR-148/-152 family, plays a key role in regulating fibrosis.^54^ MiR-148a inhibits liver fibrosis by targeting USP4,^55^ while miR-148a-3p suppresses alcoholic liver fibrosis by regulating ERBB3.^56^ In gastric cancer cells, the SMAD2 gene is a direct target of miR-148a.^57^ Notably, miR-148a-3p, delivered via hucMSC-derived extracellular vesicles, has been shown to target Hsp90b1 and prevent silica-induced pulmonary fibrosis.^58^ Based on these findings, we proposed that the delivery of miR-148a-3p to LECs by hucMSC-Exo may contribute to its anti-fibrotic effects. Through gain- and loss-of-function experiments, we found the inhibitory role of miR-148a-3p in the LECs-EMT. The inhibitory effect of hucMSC-Exo on LECs-EMT was attenuated after co-treatment with miR-148a-3p inhibitor. We have also identified PRNP as a novel target of miR-148a-3p. These findings suggest that miR-148a-3p is a key mediator of the anti-fibrotic effects of hucMSC-Exo.

From the miRNA expression profile of hucMSC-Exo, we can observe that hucMSC-Exo also contained a significant number of miRNAs that negatively regulate EMT, such as miR-22,^29^ miR-26,^28^ miR-125, miR-145, miR-21, miR-23a-3p,^41^ miR-27b,^33^ miR-182-5p.^24^ Therefore, we hypothesized that the inhibitory effect of hucMSC-Exo on LECs-EMT may result from the synergistic action of multiple molecules. However, further research is needed to determine whether other miRNAs mediate the anti-fibrotic effect of hucMSC-Exo on LECs-EMT, as the same molecule may have completely different effects on different cells or under different conditions. In addition, exosomes carry various bioactive molecules, including miRNAs, mRNA, lncRNA, DNA, proteins, and lipids.^20^ Proteins,^59^ lncRNA,^60^ and mRNA^61^ within exosomes play a role in EMT. Thus, the mechanism by which hucMSC-Exo exerts its effects may not be mediated by miRNAs alone but rather involves a combination of multiple molecules. Further research is needed to investigate these interactions.

In the regulatory network of multiple signaling pathways involved in EMT, the TGFβ/Smad signaling pathway is considered to play a crucial role.^62^ Additionally, recent studies have highlighted the important role of the ERK pathway. Activation of ERK/MAP kinase signaling contributes to and is even required for, TGFβ-induced EMT.^62,63^ ERK can phosphorylate Smad1, which is essential for the transcriptional activation of TGFβ-responsive genes.^64^ Inhibition of MEK1/2 kinase with a chemical inhibitor leads to inactivation of ERK, thereby suppressing TGFβ-induced EMT.^63^ In LECs, ERK signaling can cross-interact with canonical TGFβ/Smad signaling, which subsequently contributes to LECs-EMT.^46^ Consistent with our findings, ERK was rapidly activated in LECs-EMT.^65^ Furthermore, a specific ERK inhibitor can block the morphological changes in LECs and the TGFβ-induced upregulation of Slug.^65^ Furthermore, a study in LECs indicated that blocking TGFβ2/Smad2/3 signaling with SB431542 did not inhibit TGFβ2-induced ERK activation; however, inhibiting MEK/ERK1/2 with U0126 completely prevented TGFβ2-mediated LECs-EMT.^46^ These findings suggest that ERK activation is crucial for LECs-EMT, but it occurs independently of the TGFβ/Smad signaling pathway.

PrP^C^ is a conformational isoform of a normal glycosylphosphatidylinositol (GPI)-anchored protein that is expressed on the plasma membrane of all cells.^66^ PrP^C^ is highly conserved among mammalian species, indicating its important role in organisms.^67^ Beyond its well-known association with prion diseases, PrP^C^ has been shown to be involved in the process of EMT.^68^ For example, PrP^C^ regulates TGFβ downstream signaling^69^ and promotes EMT in cancer cells through ERK (MAPK) activation.^43^ Moreover, PrP^C^ is involved in ERK activation during the EMT process.^43,70^PrP^C^ is expressed in LECs and may play a specific role in maintaining LECs homeostasis.^45^ Our results showed the inhibitory role of miR-148a-3p in LECs-EMT and identified PRNP as a direct target gene of miR-148a-3p. Additionally, TGFβ2 treatment upregulated PRNP expression, which was associated with ERK activation. Application of MSC-Exo or overexpression of miR-148a-3p inhibited LECs-EMT, leading to the downregulation of PRNP and inactivation of ERK. Given that activation of ERK is independent of TGFβ/Smad signaling in LECs.^46^ Therefore, we speculate that PrP^C^ is involved in regulating LECs-EMT and is likely responsible for ERK activation. However, the exact role of PRNP in lens EMT and its relationship with the ERK signaling pathway require further investigation. Taken together, these findings suggest that the miR-148a-3p/PRNP/ERK cascade plays an important role in LECs-EMT.

## Conclusions

Our results demonstrated that hucMSC-Exo suppressed LECs-EMT and uncovered a novel regulatory mechanism: exosomal miR-148a-3p downregulated its target gene PRNP, leading to the inactivation of the downstream ERK signaling pathway and ultimately inhibiting EMT. These findings not only expand our understanding of the pathogenesis of fibrosis but also provide potential therapeutic strategies for the treatment of fibrotic cataracts.

## Supplementary Material

szae091_suppl_Supplementary_Tables_S1-S2

## Data Availability

The data supporting the conclusions of this article are provided in this article and its supplementary information files.
