# Supplementary material for: Exosomes containing miR-148a-3p derived from mesenchymal stem cells suppress epithelial-mesenchymal transition in lens epithelial cells
Source: Stem Cells Transl Med. 2025 Feb 26;14(2):szae091. doi: 10.1093/stcltm/szae091 (PMC11878568; doi:10.1093/stcltm/szae091)
Supplement: szae091_suppl_Supplementary_Tables_S1-S2 [file szae091_suppl_supplementary_tables_s1-s2.docx]

**Supplementary Information**

Additional file 1:

Table S1. The sequences of miR-148a-3p inhibitor, inhibitor NC, miR-148-3p mimic and mimic NC.

| has-miR-148a-3p | | |
| --- | --- | --- |
| mimic | Sense | 5’-UCAGUGCACUACAGAACUUUGU-3’ |
|  | Antisense | 5’-AUAAAGUUCUGUAGUGCACUGA-3’ |
| inhibitor | 5’-AUAAAGUUCUGUAGUGCACUGA-3’ | |
| Negative ctrl (NC) | | |
| mimic-NC | 5’-UUUGUACUACACAAAAGUACUG-3’ | |
|  | 3’-AAACAUGAUGUGUUUUCAUGAC-5’ | |
| inhibitor-NC | 5’-CAGUACUUUUGUGUAGUACAAA-3’ | |

| Gene | Primer sequence 5‘-3’ |
| --- | --- |
| ACTA-2 | Forword：CTATGCCTCTGGACGCACAACT |
|  | Reverse：CAGATCCAGACGCATGATGGCA |
| Snail | Forword：TCGGAAGCCTAACTACAGCGA |
|  | Reverse：AGATGAGCATTGGCAGCGAG |
| Vimentin | Forword：AGTCCACTGAGTACCGGAGAC |
|  | Reverse：CATTTCACGCATCTGGCGTTC |
| N-cadherin | Forword：AGCCAACCTTAACTGAGGAGT |
|  | Reverse：GGCAAGTTGATTGGAGGGATG |
| Fibronectin | Forword：CGGTGGCTGTCAGTCAAAG |
|  | Reverse：AAACCTCGGCTTCCTCCATAA |
| E-cadherin | Forword：ATTTTTCCCTCGACACCCGAT |
|  | Reverse：TCCCAGGCGTAGACCAAGA |
| PRNP | Forword：AGTCAGTGGAACAAGCCGAG |
|  | Reverse：CTGCCGAAATGTATGATGGGC |
| GAPDH | Forword：TCGTGGAAGGACTCATGACC |
|  | Reverse：AGGCAGGGATGATGTTCTGG |
| hsa-miR-21-5p | Forword：TAGCTTATCAGACTGATGTTGA |
| hsa-miR-22-3p | Forword：AAGCTGCCAGTTGAAGAACTGT |
| hsa-let-7a-5p | Forword：TGAGGTAGTAGGTTGTATAGTT |
| hsa-miR-125b-5p | Forword：CCCTGAGACCCTAACTTG |
| hsa-let-7f-5p | Forword：GCGTGAGGTAGTAGATTG |
| hsa-miR-148a-5p | Forword：TCAGTGCACTACAGAACTT |
| hsa-let-7i-5p | Forword：TGAGGTAGTAGTTTGTGCTGTT |
| hsa-miR-16-5p | Forword：TAGCAGCACGTAAATATTGGCG |
| hsa-miR-23a-3p | Forword：ATCACATTGCCAGGGATTTCC |
| hsa-miR-199a-3p | Forword：ACAGTAGTCTGCACATTGGTT |
| U6 | Forword：CTCGCTTCGGCAGCACATATACT |

Table S2. The sequences of the qRT-PCR primers.
